# Customizing intense interval exercise training prescription using the “frequency, intensity, time, and type of exercise” (FITT) principle

**DOI:** 10.3389/fphys.2025.1553846

**Published:** 2025-04-03

**Authors:** Pinelopi S. Stavrinou, Todd A. Astorino, Christoforos D. Giannaki, George Aphamis, Gregory C. Bogdanis

**Affiliations:** ^1^ Department of Life Sciences, University of Nicosia, Nicosia, Cyprus; ^2^ Department of Kinesiology, CSU-San Marcos, San Marcos, CA, United States; ^3^ School of P. E. and Sport Science, National and Kapodistrian University of Athens, Athens, Greece

**Keywords:** high intensity interval training, sprint interval training, frequency, cardiorespiratory fitness, heart rate, blood lactate concentration, metabolic, perceptual responses

## Abstract

Intense interval exercise training induces various physiological and metabolic adaptations related to performance and health. For designing a program, the F.I.T.T. principle, referring to frequency, intensity, time, and type of exercise, can be used to manipulate the level of physiological stress in the body, leading to various adaptations. Modifying these four parameters results in a wide range of interval protocols that are safe and effective for different populations including athletes and individuals with chronic diseases. In this review, we present how the manipulation of the F.I.T.T. components can alter the acute and chronic cardiorespiratory, metabolic, perceptual, and affective responses and adaptations to intense interval exercise training. From this evidence, it appears that the duration of the exercise bout and recovery interval are critical parameters for the manipulation of almost all acute responses, enabling periodization of intense interval exercise training, and promoting optimal adaptations and exercise adherence. In addition, a considerable level of adaptations may be achieved with training frequencies as low as once or twice per week and with lower than maximal intensities, adding to the feasibility of this exercise mode. Overall, by varying these parameters, the design of an intense interval exercise training program can be tailored according to the needs and abilities of each individual, and an optimized training prescription may be achieved.

## 1 Introduction

Intense interval exercise training is a collective term that includes all types of training performed intermittently at intensities higher than certain physiological thresholds, such as the lactate threshold, the maximal oxygen uptake or the critical speed/power (Buchheit and Laursen, 2013; Gibala et al., 2014; Coates et al., 2023). Even though there is a lack of standardized terminology, recent work has sub-categorized interval exercise performed at high intensity using physiological and performance indices into high-intensity interval training (HIIT) and sprint interval training (SIT) (Coates et al., 2023; Stöggl et al., 2024). The lower boundary of HIIT is demarcated by the intensity corresponding to the second lactate threshold or critical power (around 75%–85% of maximal oxygen uptake - VO_2_max or above the lower boundary of “severe” exercise domain). However, the upper boundary of HIIT is debatable, due to the lack of definition of an intensity above which an effort is considered as “sprint” (Alexander et al., 2019; Jones et al., 2019; Coates et al., 2023). Thus, most authors consider as HIIT exercise performed at an intensity up to the speed or power corresponding to the maximal oxygen uptake (vVO_2_max), while others include as HIIT higher intensities up to 30% of the “anaerobic speed or power reserve” (ASR), which is defined as the difference between vVO_2_max and maximum speed or power (Sandford et al., 2021). Thus, the lower boundary of sprint interval exercise or “SIT” is somehow uncertain due to a poor definition of a “sprint threshold”. Therefore, some authors consider as SIT exercise above 100% of the intensity corresponding to VO_2_max, others define as SIT exercise at intensities above 130%–150% of maximum aerobic speed, and others include only the “all-out” efforts (Gibala et al., 2014; Gillen et al., 2016; Costa et al., 2018; Taylor et al., 2019; Sabag et al., 2022; Coates et al., 2023; Hall et al., 2023).

Intense interval exercise training can also be sub-categorized based on bout duration into long and short-duration efforts. The boundaries of short bout duration may be arbitrarily set between 6 and 60 or 90 s, while bout durations longer than 60–90 s (e.g., 2–4 min), may be termed “long bouts”. Of course, there is a relationship between intensity and bout duration. However, short bouts (10–30 s) may also be prescribed at intensities around 100% VO_2_max or maximal heart rate (HRmax), usually with equal recovery durations (i.e. 15 s–15 s) (Dellal et al., 2010), while this intensity may also be used for longer bouts (e.g., 4 min) (Wisløff et al., 2007; Tjønna et al., 2013). In most cases, the total exercise duration of HIIT at such intensities is from 6 to 16 min (not including recovery) (Ramos et al., 2017; Stavrinou et al., 2018), while in SIT, the total exercise duration is between 40 s and 3 min (Metcalfe et al., 2012; Bogdanis et al., 2013). In another available classification, HIIT can be further sub-categorised into low- and high-volume HIIT according to the cumulative interval duration. Low-volume HIIT (LV-HIIT) protocols have been defined as those that accumulate <15 min of time spent during the high intensity intervals, with all other HIIT protocols being defined as high-volume HIIT (Sabag et al., 2022).

There is growing scientific evidence regarding the positive effects of HIIT and SIT on physiological indices such as cardiorespiratory fitness (Stavrinou et al., 2018), hormonal responses (Bogdanis et al., 2022b), glucose regulation (Khalafi et al., 2022), fat oxidation (Tsirigkakis et al., 2021), brachial artery vascular function (Ramos et al., 2015), antioxidant status (Bogdanis et al., 2013), blood pressure (Costa et al., 2018), and decreased abdominal and visceral fat mass (Maillard et al., 2018). The fact that such adaptations may be equal or even greater compared with moderate-intensity continuous training (Ramos et al., 2015; Keating et al., 2017; Mattioni Maturana et al., 2021), makes intense interval training an efficient exercise strategy. Furthermore, this type of training appears to be a viable intervention also to improve various psychological indices such as mental wellbeing, depression severity and perceived stress (Martland et al., 2022), and in several cases promotes favorable affective responses (Astorino and Thum, 2018) and exercise enjoyment (Stavrinou et al., 2019). These perceptual responses to intense interval exercise are of great importance since they may promote habitual physical activity behaviour and may predict future exercise adherence (Rhodes and Kates, 2015; Stavrinou et al., 2019).

Due to these substantial health and fitness-related benefits, it is unsurprising that the popularity of HIIT and SIT has increased in recent years. However, a single, optimal protocol remains elusive. For this mode of exercise to be widely applicable, intense interval exercise training should be tailored to adults having different needs, abilities, desired adaptations, or even health status and disease (Wisløff et al., 2007; Fex et al., 2015; Taylor et al., 2019). Current exercise prescription guidelines for developing and maintaining fitness and health in various populations are based on the F.I.T.T. principle, namely, Frequency (number of sessions per week), Intensity (level of exertion during exercise and recovery), Time (duration of exercise bout, recovery interval duration between bouts and sets and total session duration and time-structure), and Type (mode of exercise and recovery, as well as the use of different types of loading, such as body mass, external resistances, etc.) (Garber et al., 2011; Bull et al., 2020). The design of any intense interval exercise training program should consider the components of the F.I.T.T. principles, as these elements are interconnected and influence each other. The combination of these components produces a variety of exercise protocols, making interventions of interval training highly diverse. Understanding how the manipulation of each component can impact the psychophysiological responses, and the magnitude of training-induced adaptations is essential to effectively prescribe intense interval exercise training. Prior systematic reviews and meta-analyses have comprehensively examined the effects of HIIT/SIT on changes in various physiological and psychological responses (Maillard et al., 2018; Khalafi et al., 2022; Metcalfe et al., 2022), yet no review has simultaneously examined how changing these four F.I.T.T elements may impact the acute or adaptive response. This perspective review provides a comprehensive alternative guide to solving the programming puzzle of intense interval exercise training for populations with diverse training backgrounds, fitness levels, and health statuses—an essential resource for practitioners utilizing HIIT as a training modality. Therefore, this review aims to explore the effects of manipulating the F.I.T.T. components for the development of individualized and specifically targeted HIIT/SIT prescriptions, with an overall goal of optimizing the adaptive response to training.

## 2 Frequency

In most training studies, intense interval exercise training was performed at a frequency of three sessions per week which induces various favourable cardiorespiratory, metabolic, body composition and psychological adaptations (Little et al., 2011; Gillen et al., 2016; Stavrinou et al., 2018). However, few studies have examined the effects of a lower or higher frequency on different physiological and psychological indices.

### 2.1 Cardiorespiratory adaptations

The study by Stavrinou et al. (2018) compared the efficacy of LV-HIIT with low and moderate training frequency (2 and 3 times per week) on changes in cardiorespiratory fitness in healthy inactive adults. Following 8 weeks of training, VO_2_peak was significantly and similarly increased in response to both low (by 3.5 mL·kg^-1^·min^-1^; 10.8%) and moderate (by 4.5 mL·kg^-1^·min^-1^; 13.6%) training frequency. Another study examined the effect of training frequency (2, 3 or 4 sessions/week) on changes in VO_2_max in response to 6 weeks of reduced-exertion sprint interval training (RESIT; 2 × 10–20 s all-out sprints interspersed in 10 min of unloaded cycling) in inactive individuals (Thomas et al., 2020). Their results showed that performing two RESIT sessions per week is sufficient to significantly improve VO_2_max, and that performing three or four sessions per week does not significantly augment the magnitude of improvement. Even training once a week with LV-HIIT (12 × 1 min bouts, 90% HRreserve) was found to increase estimated VO_2_max in comparison with a non-exercising control group after 8 weeks (Chin et al., 2020). This result supports data showing that VO_2_max and ventilatory threshold significantly increased by 13% and 21%, respectively, in healthy young males who underwent interval training (three bouts of cycling at 80% of maximum work rate until volitional fatigue) once per week for 3 months (Nakahara et al., 2015). In addition, an 18% increase in left ventricular posterior wall thickness was found, with the authors suggesting that the exercise stimulus necessary to induce cardiac morphological changes in nonathletes may not require high frequency and high-volume training if an adequate work intensity is ensured (Nakahara et al., 2015). Similarly, in older adults, performing 6 s bouts of SIT 1 or 2 days/wk for 8 weeks elicits similar improvements in aerobic capacity (5%) (Adamson et al., 2020). These findings are supported by a recent meta-analysis which demonstrated no significant effect of the number of HIIT sessions performed per week on peak VO_2_ improvement in the general population (Edwards et al., 2023). Therefore, even though in the previously mentioned studies the protocols were not work-matched, it has been suggested that the accumulation of total HIIT volume, through different training frequencies, may influence the time course, but not the overall magnitude, of cardiorespiratory fitness adaptations (Stavrinou et al., 2018).

On the other hand, it was recently shown that six LV-HIIT sessions (10 × 60 s at 100% VO_2_max interspersed with 75 s of low-intensity cycling) performed twice a day over 5 days improved VO_2_max and endurance capacity (Atakan et al., 2021). The authors suggested that these rapid improvements are associated mainly with peripheral adaptations. However, the long-term effects of this protocol are unknown. When young healthy participants performed 24 sessions of high-volume HIIT (4 × 4 min intervals at 90%–95% HRmax) at either high (eight sessions/week) or moderate (three sessions/week) frequency (Hatle et al., 2014), results showed that the overall increases in VO_2_max were similar. However, in high-frequency HIIT there was a delayed increase in VO_2_max which occurred during the detraining period. The results suggest that high-frequency HIIT may induce fatigue and highlight the necessity of sufficient rest to adapt from such a heavy exercise training programme. Furthermore, it is evident that older adults require longer than 3 days to recover from a single bout of HIIT (Herbert et al., 2015), which has to be considered when designing HIIT protocols for this population.

### 2.2 Changes in metabolic indices and body composition

Less data is available concerning how altering training frequency may affect other parameters besides cardiorespiratory fitness. Regarding glucose regulation, no effect of low LV-HIIT frequency on fasting glucose, insulin, or HbA1c was found in a previous study (Stavrinou et al., 2018). However, the participants were healthy non-obese young adults who had minimal capacity to change these outcomes with training. Similarly, fasting glucose did not change in response to either once or twice sessions per week of SIT in older adults (Adamson et al., 2020) or LV-HIIT in overweight/obese adults (Chin et al., 2020). However, when oral glucose tolerance tests were conducted, the study by Stavrinou et al. (2018) observed lower plasma insulin at the end of the test in both training groups (two and three sessions/week) compared with baseline. Interestingly, a decrease in 2-h blood glucose and glucose area under the curve was found when older adults performed twice-weekly but not once-weekly SIT (Adamson et al., 2020). Similarly, the glucose area under the curve was significantly reduced by 6% following 8 weeks of twice/week SIT in middle-aged adults (Adamson et al., 2014). As for changes in blood lipids, performing LV-HIIT thrice but not twice per week resulted in a significant reduction of total cholesterol and LDL-C in inactive adults (Stavrinou et al., 2018), while no change in HDL-C or triglycerides occurred following either low or moderate HIIT frequency. Nevertheless, the impact of intense interval exercise training on lipid profile is an area that requires further examination as illustrated by the contradictory results found from meta-analyses (Batacan et al., 2017; Martland et al., 2020).

Prior reports show that HIIT (Talanian et al., 2007) and SIT (Burgomaster et al., 2008; Astorino et al., 2011) increase exercise fat oxidation derived from respiratory exchange ratio, which is likely related to increases in activities of β-hydroxyacyl-CoA dehydrogenase (β-HAD) and citrate synthase (Talanian et al., 2007; Burgomaster et al., 2008). A significant inverse dose-response relationship has been reported in a recent meta-analyisis between the number of interval exercise sessions per week and the increases in fat oxidation; however, this result should be interpreted with caution because of the low number of studies in which participants exercised less or more than three times per week (Atakan et al., 2022). In one study in recreationally active males, fat oxidation during submaximal exercise significantly increased following two sessions of HIIT per day every other day for 5 days but not following six sessions of HIIT over 2 weeks (Atakan et al., 2021).

Regarding changes in body composition, Stavrinou et al. (2018) showed that although body mass remained unchanged following 8 weeks of LV-HIIT, performing training two or 3 days per week significantly reduced waist circumference and increased thigh lean cross-sectional area, indicating an increase in leg muscle mass. However, total body fat and trunk fat were significantly decreased only in the group that trained three times per week. In contrast, another study in overweight or obese individuals showed that one session of HIIT weekly significantly reduced body fat mass and trunk fat mass when compared with control (Chin et al., 2020). These findings support a study in lifelong sedentary aging men in which a significant reduction in body fat with a concomitant increase in lean body mass were observed following one session of LV-HIIT every 5 days for 6 weeks (Sculthorpe et al., 2017).

### 2.3 Psychological responses

There is evidence suggesting that LV-HIIT performed by previously inactive individuals on two or 3 days per week induces high levels of perceived enjoyment and intention to implement high-intensity exercise in the future (Stavrinou et al., 2019). The same study demonstrated an increase in the participants’ habitual physical activity 2 months after the cessation of the HIIT intervention, highlighting the positive influence of HIIT on promoting sustainable physical activity participation, irrespective of training frequency. Another study observed higher adherence rates when performing LV-HIIT once or twice per week compared to 3 days per week, suggesting that the lower HIIT frequencies are more feasible for initiating exercise training in overweight and obese population (Chin et al., 2020). On the other hand, a recent meta-analysis showed greater improvements in perceived stress when HIIT was conducted at least twice weekly, compared to a lower frequency (Martland et al., 2022).

### 2.4 Conclusion for manipulating frequency

The results of these studies indicate that cardiovascular adaptations may occur even after a small and less frequent number of HIIT/SIT sessions. Less and contradictory data regarding the effects of low HIIT frequency on changes in body composition exist in the literature, likely due to the different populations studied. Regarding changes in metabolic indices, the existing literature indicates that whole-body glucose metabolism can be improved even with twice-weekly HIIT/SIT. On the contrary, minimal available data show that low-frequency HIIT/SIT does not induce favorable blood lipid changes, suggesting that a training volume threshold should be surpassed to elicit positive changes. Regarding psychological indices, low HIIT/SIT frequency seems to be viewed as enjoyable and may promote future adherence to physical activity. Taken together, even though there is inadequate data to derive certain conclusions for all parameters, the available results are promising in that less frequent intense interval exercise training sessions may still induce positive adaptations. This is crucial as moderate/high training frequency may not be feasible for individuals with time constraints or for clinical and aging populations. Therefore, performing low-frequency intense interval exercise training might provide a time-efficient and practical exercise strategy that can be easily implemented in daily life to improve health and performance. Nevertheless, additional studies examining the effects of varying frequencies of HIIT or SIT on those parameters are needed, to draw robust conclusions.

## 3 Intensity

Examination of HIIT and SIT studies reveals divergent intensities within each category of interval training which may lead to different physiological, metabolic and perceptual/affective responses and adaptations. In addition, there is a lack of standardization of various HIIT and SIT protocols. Thus, it is difficult to compare the results of interval training programs having different intensities due to inconsistent exercise protocols (Viana et al., 2018). For example, setting exercise intensity using a percentage of maximum aerobic speed or maximal workload (e.g., 105% of the velocity at VO_2_max) will not always induce the desired intensities. First, there is a lag in the HR response which means that the entire exercise bout duration is not spent at the desired intensity (Acala et al., 2020), although VO_2_ increases faster (Midgley et al., 2007). Furthermore, it is apparent that the average HR and VO_2_ attained during the session depend on the duration and intensity of the exercise bout and the recovery (Dellal et al., 2010; Bogdanis et al., 2022c).

For instance, previous studies show the effectiveness of very short intense bouts (i.e. 10 s–10 s, 15 s–15 s) around the critical velocity as a means to improve aerobic fitness (Billat et al., 2001). However, these widely used protocols are insufficient to induce a high HR response (Dellal et al., 2010). This is because the exercise intensity corresponding to VO_2_max does not elicit HR or VO_2_ values above 85% of HRmax, possibly due to the interval nature of the protocols.

### 3.1 Cardiorespiratory responses and adaptations

A very recent umbrella review showed that intense interval exercise training significantly increased cardiorespiratory fitness in adults compared to non-exercise control with a standardized weighted mean difference of 3.2–5.5 mL/kg/min (Poon E. T. et al., 2024). Another meta-analysis demonstrated a statistically significant increase in peak VO_2_ following intense interval exercise training by 3.9 mL min^−1^ kg^−1^. Subgroup analyses showed significant improvements following both aerobic interval training (4 × 4-min protocols; 3.77 mL min^−1 ^kg−^1^) and SIT (3.25 mL min^−1^ kg^−1^) with no significant difference between them (Edwards et al., 2023). Three other meta-analyses have shown that there is no significant difference in improvements in VO_2_max between HIIT and SIT (Rosenblat et al., 2020; 2022; de Oliveira-Nunes et al., 2021). In another study, interval cycling performed for 12 weeks led to significantly similar improvements in VO_2_max in sedentary young women whether intensities were moderate (60%–80% PPO) or more rigorous (80%–90% PPO) (Astorino et al., 2013), however, the magnitude of improvement in VO_2_max was greater early on in higher intensity compared to lower (Astorino et al., 2013; Boyd et al., 2013). In contrast, another meta-analysis in overweight/obese adults indicated that HIIT protocols at an intensity of 7–7.9 METs (i.e., ∼75%–85% VO_2_max or ∼85–90% HRmax) may be more effective in increasing cardiorespiratory fitness than lower intensity protocols (6–6.9 METs) (Wang et al., 2021). Interestingly, two recent studies (Helgerud et al., 2023; Hov et al., 2023) in aerobically well-trained men and women demonstrated that VO_2_max increased significantly more following HIIT (4 × 4 min at ∼95% maximal aerobic speed with 3 min active breaks) than two SIT protocols (8 × 20 s at ∼150% maximal aerobic speed with 10 s passive breaks; 10 × 30 s session at ∼175% maximal aerobic speed with 3.5 min active breaks). The authors argued that aerobic intensity (i.e., accumulated time spent ≥90% VO_2_max), and not overall intensity (% of maximal aerobic speed), seems paramount for enhancing VO_2_max. Both central cardiovascular function and peripheral O_2_ extraction mediate the increase in VO_2_max observed in response to intense interval training (Daussin et al., 2008; Bostad et al., 2021; Astorino et al., 2022). Nevertheless, the mechanisms that may underlie improvements in VO_2_max can vary in each interval type. A recent systematic review and meta-analysis reported that increases in VO_2_max demonstrated with intense interval training are attendant with increases in central O_2_ delivery with little contribution from changes in hematocrit, blood volume, or plasma volume (Astorino et al., 2022). The authors also suggested that there may be different time courses for the cardiac output response to interval training between SIT and HIIT. Comparing HIIT and SIT, a meta-analysis (Rosenblat et al., 2022) showed that only HIIT led to significant improvements in cardiac function as it may produce greater increases in maximal stroke volume and cardiac output compared with SIT. However, SIT may be the optimal method of interval training to improve peripheral factors, as SIT had a greater influence on angiogenesis versus HIIT, although studies are limited. According to the same meta-analysis, both HIIT and SIT increased maximal citrate synthase activity, while changes in other peripheral measures (capillary density, mitochondrial respiration) only occurred with SIT (Rosenblat et al., 2022). Both HIIT and SIT studies have shown increased arteriovenous difference following training (Daussin et al., 2008; Raleigh et al., 2018; Bostad et al., 2021). Regarding the role of high-intensity exercise in promoting mitochondrial adaptations, conflicting findings in the literature exist, and a consensus has not been reached (Bishop et al., 2019). It has been reported that training volume may be a critical factor affecting changes in mitochondrial content; whereas, relative exercise intensity is an important determinant of changes in mitochondrial respiratory function (Granata et al., 2018). In contrast, another review reported that exercise intensity mediates the exercise-induced adaptations in mitochondrial content (MacInnis and Gibala, 2017). Recently, it has been suggested that HIIT at a relative exercise intensity ≥90% of maximal power output provides the greatest absolute increase in mass-specific mitochondrial respiration; whereas, all-out SIT appears to be the most efficient type of exercise to improve mitochondrial respiratory function in terms of total training volume and/or time (Granata et al., 2018). Nevertheless, further research and the use of more advanced methodologies are required to explain these discrepancies.

Furthermore, the physiological effects may vary due to different exercise formats, even when exercise at the same intensity according to vVO_2_max is performed. For example, HR, blood lactate, and rating of perceived exertion (RPE) were significantly higher in soccer players when intermittent exercise at the same intensity was performed in shuttle format with directional changes compared with traditional in-line running (Dellal et al., 2010). In addition, the duration and intensity of recovery may also impact the physiological responses to intense interval exercise training. Early studies using SIT suggested that active recovery rather than passive recovery is preferred in order to increase muscle blood flow, to decrease blood lactate concentration and accelerate phosphocreatine resynthesis (Bogdanis et al., 1996). However, in short bouts of HIIT, such as the 15 s–15 s format, it has been reported that active recovery reduces time to exhaustion and thus is detrimental to performance (Dupont et al., 2004). This result is explained by the higher metabolic demand of active compared with passive recovery and the lower oxygen availability for recovery of phosphocreatine (Dupont et al., 2004).

### 3.2 Changes in metabolic indices and body composition

Prior studies comparing acute LV-HIIT and SIT protocols found dissimilar metabolic perturbation between regimens, with the SIT protocol eliciting higher blood lactate concentrations indicating a greater reliance on nonoxidative metabolism (Wood et al., 2016; Astorino and Vella, 2018). A review reported that completion of interval training increased fat oxidation in approximately 50% of studies, with the frequency of increment being higher in response to studies using HIIT compared to SIT (Astorino and Schubert, 2018). Similarly, a significant pooled effect of HIIT on fat oxidation was reported in a meta-analysis; whereas, the effect of SIT was not significant, potentially due to a small number of SIT studies (Atakan et al., 2022). Regarding body adiposity, meta-analyses have shown similar reductions in body fat loss (%) between HIIT and SIT (Keating et al., 2017; Mattioni Maturana et al., 2021; Poon E. T.-C. et al., 2024). In contrast, according to another meta-analysis, high intensities (above 90% of HRpeak) seem more likely to reduce whole-body adiposity, and lower intensities are more successful in reducing abdominal and visceral fat mass (Maillard et al., 2018).

Various forms of ΗΙΙΤ have also been found to be effective for the improvement of glucose regulation by reducing insulin resistance (Jelleyman et al., 2015) and postprandial glucose and insulin area under the curve (AUC) (Khalafi et al., 2022). In a recent meta-analysis, a subgroup analysis by type of intense interval exercise training revealed a significant reduction in glucose AUC for SIT, while for longer interval HIIT, the reduction approached statistical significance. These beneficial effects were shown primarily in participants with impaired glucose. On the contrary, other meta-analyses found no effects of interval intensity on insulin resistance, fasting glucose, fasting insulin, or HbA1c improvements (Jelleyman et al., 2015; Mattioni Maturana et al., 2021). Regarding blood lipid profile, SIT was found to be superior to HIIT in attenuating LDL and total cholesterol, while no differences were found for triglycerides and HDL (Mattioni Maturana et al., 2021).

### 3.3 Psychological responses

It has been reported that as exercise intensity increases and approaches maximal capacity, a decline in pleasure occurs (Ekkekakis et al., 2011). Particularly, pleasure is reduced above the ventilatory or lactate threshold (Ekkekakis et al., 2011), which is characteristic of HIIT and especially SIT leading to marked blood lactate accumulation and an integrated psychobiological stress response. Therefore, there is debate whether interval exercise at supramaximal intensities is practical or feasible to complete, especially for sedentary populations.

Prior work (Astorino and Vella, 2018) documents an intensity-dependent relationship between affect and intensity during interval exercise, as supramaximal intensities [130% of peak power output (PPO)] elicited a larger decline in affect compared to submaximal intensities (85% PPO). The authors found that blood lactate accumulation mediated the affective responses to exercise. On the other hand, in this study neither enjoyment nor rating of perceived exertion (RPE) differed between sprint or high-intensity interval exercise. In another acute study, moderately active young men completed three high-intensity cycling protocols (SIT: 4 × 30 s all-out sprints; Tabata: 7 × 20 s at 170% VO_2_max; and HIIT: 10 × 60 s at 90% HRmax) (Follador et al., 2018). The Tabata protocol elicited the highest RPE responses, and the most post-exercise aversive affect among the trials, while the 10 × 60 s trial was the least strenuous and promoted the most positive session-affective responses. However, it is important to highlight that the intensity of exercise *per se* is not always the criterion for the psychological responses during HIIT, especially in short HIIT bouts. As stated above, there is a delay in the cardiorespiratory responses following the initiation of the bout (Midgley et al., 2007), possibly causing more positive perceptual responses with shorter bouts. Thus, the effects of the manipulation of exercise intensity should be examined without changing the bout duration or total exercise time (total exercise time and total recovery time duration).

For instance, it seems that when the duration of SIT bouts is reduced, the difference in affect between SIT and HIIT diminishes, despite the “all-out” work intensity (more on this subject under the “time” section). There is evidence that among inactive adults, affective responses to a 3 × 20-s “all-out” SIT protocol are comparable to affective responses to a 10 x 1-min HIIT protocol (Stork et al., 2018). In addition, equal levels of post-exercise enjoyment and preferences were reported. In a training study comparing thrice-weekly SIT (80 × 6 s “all-out” cycling interspersed with 9 s rest), and HIIT (4 min cycling at 90% VO_2_peak followed with 3 min recovery for ∼60 min) with equivalent mechanical work, enjoyment immediately after the completion of the session was equal in overweight/obese young women (Hu et al., 2021). Similar to the previous study (Stork et al., 2018), the authors suggested that the shortened sprint duration in SIT might lessen the magnitude of physiological stress and mitigate the adverse feelings caused by the supramaximal intensity.

### 3.4 Conclusion for manipulating intensity

HIIT and SIT protocols promote similar gains in cardiorespiratory fitness, possibly due to different contributions of central and peripheral mechanisms. The results regarding the differences in metabolic adaptations and body composition changes following HIIT or SIT are less clear. Higher intensities induce increased metabolic disturbances such as greater blood lactate concentration and physiological perturbations (such as decreases in plasma volume and blood pressure) (Bogdanis et al., 1996) and may elicit more aversive responses and higher levels of perceived fatigue. However, this could be significantly modulated if the duration of the bouts is reduced.

## 4 Time

Various responses and adaptations to intense interval exercise training may also be manipulated by altering different “time” variables, such as the duration of exercise bouts and the rest period interspersing them, the number and bouts and sets, and the total session and training duration. These changes in the time-structure of training may induce large variations in physiological and metabolic responses, despite total training time remaining similar. The fluctuations of physiological parameters during high-intensity exercise are associated with the degree of cardiorespiratory and metabolic adaptations and in turn, the perceptual responses (Daussin et al., 2008; Nicolò et al., 2014). Moreover, metabolic fluctuations during intermittent exercise may affect various signaling cascades to regulate mitochondrial biogenesis (Combes et al., 2015) and improve glucose control (Little et al., 2011; Sabag et al., 2022).

### 4.1 Bout and rest duration

#### 4.1.1 Cardiorespiratory responses and adaptations

**FIGURE 1:**
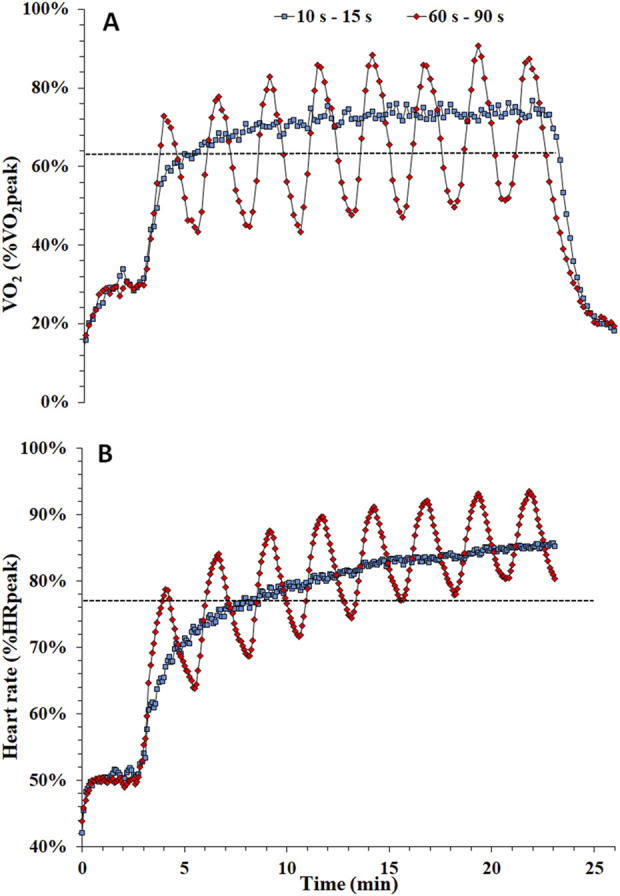
Time course of oxygen uptake [expressed as percentage of peak oxygen uptake - %VO_2_peak, panel **(A)**] and heart rate [expressed as percentage of peak heart rate - %HRpeak, panel **(B)**] during a protocol consisting of 48 × 10 s bouts interspersed by 15 s recovery (10 s–15 s, represented by blue squares) and 8 × 60 s bouts interspersed by 90 s recovery (60 s–90 s, represented by red diamonds). Dotted line in A and B represents the lactate threshold [Adapted from (Bogdanis et al., 2022c)].

A recent study demonstrated that physiological responses to acute HIIT may be manipulated by changing the bout duration while maintaining all other parameters identical (i.e., exercise intensity, total exercise and recovery duration, exercise-to-recovery ratio, total mechanical work) (Bogdanis et al., 2022c). In that study, the cardiorespiratory responses of three isoenergetic HIIT cycling protocols were compared, with the intensity of all protocols alternated between 100% and 15% of PPO for either 10, 30 or 60 s, with a work-to-rest ratio of 1:1.5. Data showed that reducing bout duration from 60 s to 10 s attenuated the fluctuations and peak values of all cardiorespiratory parameters (i.e., VO_2_, HR, pulmonary ventilation, and respiratory frequency), although the average VO_2_ and HR did not differ between protocols. It is noteworthy that in the 10 s bouts, VO_2_ did not fluctuate throughout exercise and was sustained at a value 10%–15% above the lactate threshold (see Figure 1), suggesting that protocols involving shorter duration bouts may serve as a means of training close to the lactate threshold, although with a much higher neuromuscular load. Similar findings were reported in a study involving rowing-based work-matched HIIT at lower intensity (85% PPO) (Astorino et al., 2023). The two protocols (8 × 60 s vs 24 × 20 s) displayed discrepant physiological responses with higher peak VO_2_ and HR values in response to 60 s compared to 20 s exercise bouts, despite similar mean values.

**FIGURE 2:**
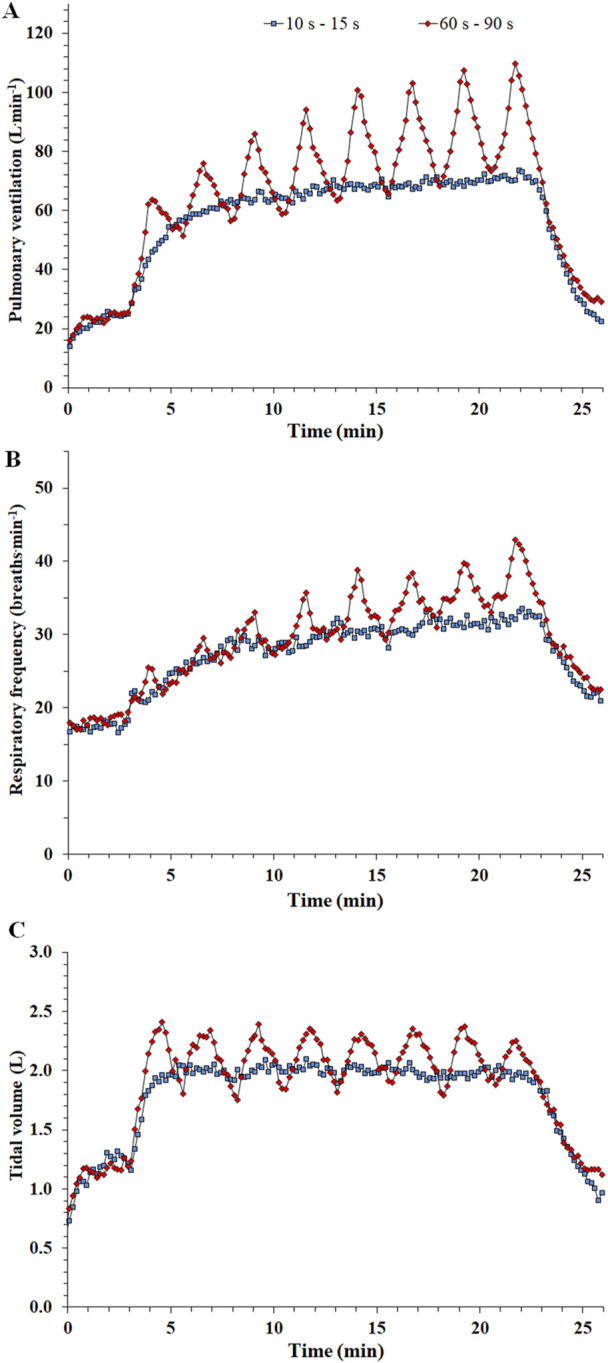
Time course of pulmonary ventilation [panel **(A)**], respiratory frequency [panel **(B)**], and tidal volume [panel **(C)**] during a protocol consisting of 48 × 10 s bouts interspersed by 15 s recovery (10 s–15 s, represented by blue squares) and 8 × 60 s bouts interspersed by 90 s recovery (60 s–90 s, represented by red diamonds) [Adapted from (Bogdanis et al., 2022c)].

In a training study comparing two work-matched programs with different bout durations and equal intensity (48 × 10 s with 15 s of recovery or 8 × 60 s with 90 s of recovery at 100% PPO), similar HR responses attaining 80–90 %HRpeak were found between protocols during the last 10 minutes of exercise (Tsirigkakis et al., 2022). Furthermore, training elicited a reduction in HR during exercise for both protocols, possibly attributed to central adaptations. Other studies in inactive and active adults comparing protocols with different bout durations showed similar increases in VO_2_max following training (Yamagishi and Babraj, 2017; Tsirigkakis et al., 2021). However, whether this similar result is caused by a plateau of changes in VO_2_max or a lack of further adaptations due to an overreaching effect in the longer bout protocol remains to be investigated. Notably, the main respiratory parameters, i.e., tidal volume, breathing frequency and pulmonary ventilation are heavily affected by bout duration (Bogdanis et al., 2022c). For example, pulmonary ventilation was 50% higher when the exercise bout duration was 60 s, compared with a 10 s bout duration (see Figure 2). This large increase in ventilation comes both from a higher tidal volume and respiratory frequency (Figure 2), which has significant implications when applying HIIT in people with chronic obstructive pulmonary disease and other respiratory problems (Sawyer et al., 2020).

Concerning SIT studies, since it has been argued that the traditional SIT protocol consisting of 4–6 x 30-s “all-out” bouts is less tolerable and not time-efficient, some researchers have opted to shorten the duration of the sprint bout while keeping the supramaximal intensity (Metcalfe et al., 2016; Nalçakan et al., 2018; Stork et al., 2018; Hu et al., 2021). There is convincing evidence that RESIT (e.g., 2 × 20 s cycling sprints) can enhance VO_2_max in a variety of populations and to a similar extent as other high-volume SIT protocols (Metcalfe and Vollaard, 2024), making it a feasible approach for improving fitness. Similar improvements were found with stair-climbing sprints, where performing 3 × 20-s stair climbing efforts, 3 days per week for 6 weeks increased VO_2_peak by 12% (Allison et al., 2017). However, further reducing sprint duration in the RESIT protocol from 20 s to 10 s seems to lessen improvements in VO_2_max (Nalçakan et al., 2018). Therefore, it has been suggested that sprint duration in SIT protocols can be reduced to a point without attenuating the associated health benefits (Vollaard and Metcalfe, 2017). On the other hand, the meta-regressions conducted in a recent review demonstrated increased effectiveness with longer (>20 s) compared with shorter sprints (<10 s) across physical performance outcomes (Hall et al., 2023). According to the authors, these results could be attributed to the greater oxidative contributions to ATP turnover because of phosphocreatine depletion and glycolytic inhibition, and to the increased number of muscular contraction cycles that lead to greater disturbances of the metabolic environment.

Lately, studies have adopted the dispersed or exercise snacks protocol as a novel approach to increase cardiorespiratory fitness and reduce the negative impact of sedentary behaviour on cardiometabolic health (Jenkins et al., 2019; Little et al., 2019). Exercise snacks are defined as isolated ≤1-min bouts of vigorous exercise performed periodically throughout the day (Islam et al., 2022). Existing studies indicate the potential value of exercise snacks as an additional tool for increasing physical activity among the general population. For example, sprint snacks involving 3 × 20-s “all out” cycling bouts separated by 1–4-h rest, led to significant improvements in VO_2_peak, peak power output, and 150 kJ cycling time trial performance that were comparable to the benchmark of low-volume SIT (Little et al., 2019).

Regarding the time structure of a HIIT/SIT session, work-to-rest ratio manipulation may affect the physiological responses in different ways. Longer rest intervals can induce adequate physiological and metabolic recovery (i.e., phosphocreatine replenishment, higher reoxygenation of myoglobin, lactate clearance). Compared with shorter recovery, longer recovery can facilitate higher workloads in subsequent exercise bouts when work intensities are not predefined, whilst potentially lowering the aerobic stimulus of the training session (Schoenmakers et al., 2019). On the other hand, reduced recovery may elicit a greater aerobic challenge due to lower PCr replenishment and decreased glycolytic contribution to energy supply (Bogdanis et al., 1995). This was confirmed by a recent SIT meta-analysis showing that work-to-rest ratios with shorter rest periods were more effective for enhancing aerobic outcomes, whereas ratios with longer rest periods were more effective for enhancing anaerobic outcomes (Hall et al., 2023).

#### 4.1.2 Changes in metabolic indices and body composition

Previous acute HIIT studies demonstrate that when work is matched, longer exercise bouts (≥60 s) elicit higher blood lactate concentration than shorter bouts (<60 s) (Bogdanis et al., 2022c; Astorino et al., 2023), indicating a greater reliance on glycolysis and higher metabolic disturbances. Similar to longer work bouts, the higher work-to-recovery ratio also seems to activate anaerobic glycolysis to a greater extent (Myrkos et al., 2022). Furthermore, an augmentation of post-exercise blood glucose in response to longer bouts (Bogdanis et al., 2022a), possibly due to a greater counterregulatory hormone response (Peake et al., 2014). A previous study comparing two intermittent exercise protocols with equal total work duration but different bout duration (6 vs 24 s) showed lower fat oxidation concurrent with accelerated carbohydrate metabolism, increases in lactate and pyruvate and reduced muscle O_2_ availability with proportionately longer work and recovery period duration (Christmass et al., 1999). The results of another study comparing modified SIT protocols with different work bout (5–30 s) and recovery period (40–240 s) durations but having identical total exercise time, recovery duration, and the work/recovery ratio indicated that modified SIT protocols with shorter work bouts enhanced exercise energy expenditure without compromising postexercise energy expenditure, though longer SIT bouts resulted in greater fat utilization in the postexercise period (Islam et al., 2017). In a training study comparing two LV-HIIT protocols with either long (8 × 60 s) or short (48 × 10 s) exercise bouts at 100% PPO in obese men, results showed a considerable increase in fat oxidation over a wide range of exercise intensities, including a large increase in peak fat oxidation, which was shifted to higher exercise intensity similarly in both HIIT protocols (Tsirigkakis et al., 2021). The lack of differences between the two protocols was attributed to the equal energy expenditure per session.

In addition, these data exhibited a significant decrease in total and trunk fat mass irrespective of protocol as well as enhanced leg lean mass (Tsirigkakis et al., 2021). A meta-analysis of thirty-six studies with 1,130 participants revealed that a shorter exercise bout duration (≤60 s) and shorter rest (≤90 s) period are more effective for fat mass reduction than a longer bout duration (Khodadadi et al., 2023). Also, a short interval duration of ≤60 s significantly increased fat-free mass while a longer interval (>60 s) had no significant effect.

#### 4.1.3 Psychological responses

In previous non work-matched studies results showed that cardiorespiratory and metabolic responses during HIIT are linked with perceptual and affective responses (Nicolò et al., 2014; Astorino and Vella, 2018). In addition, when the protocols were work-matched (Bogdanis et al., 2022c; Astorino et al., 2023), the greater cardiorespiratory and metabolic disturbances attendant with long-duration intervals (60 s) elicited higher RPE and more aversive post-exercise responses. This is because the cardiorespiratory and metabolic responses to HIIT of the same intensity and total exercise and rest duration may be mediated by changing bout duration. Even while exercising at a maximal intensity (100% PPO) and maintaining the same total work, lowering the bout duration can diminish the magnitude of perceptual strain (Bogdanis et al., 2022c).

Regarding the long-term effects of HIIT with different bout durations, a recent study in obese men compared two work-matched protocols with the same intensity (100% PPO) and total duration (20 min) yet used long (60 s) or short (10 s) efforts (Tsirigkakis et al., 2022). Results showed that 8 weeks of HIIT decreased blood lactate concentration only in the group performing shorter bouts. Furthermore, participants completing the longer exercise bouts showed no significant reductions of RPE and a significant decrease in affective valence from pre-to post-training, while affective responses were improved in response to the short exercise bouts (10 s). It has been argued that when studying affective responses to exercise, the definition of “intensity” must reference homeostatic perturbations (Ekkekakis et al., 2024). However, as demonstrated, the negative affective responses that can arise from a high-intensity exercise or training can be manipulated by modifying bout duration, even when intensity is equally high. Therefore, shorter bouts of HIIT could be an appropriate selection when prescribing interval exercise to unfit or previously inactive individuals, on the basis that they may be perceived to be more palatable and promote future exercise participation.

It also appears that perceptual responses are also impacted by sprint duration in SIT protocols. As mentioned earlier, in light of the potential for classical SIT protocols to cause negative affective responses, more recent research has shifted toward studying the effects of protocols with a lower number of sprints and/or reduced sprint duration in order to make the training sessions more time-efficient, less strenuous, and in turn more applicable to the largely sedentary population (Stork et al., 2018; Hu et al., 2021; Metcalfe et al., 2022). The change in affect during SIT can be modified by the sprint duration, as shown in a recent meta-analysis that presented a more pronounced decline in affective valence (per sprint) observed for 15–20 and 30 s sprints compared with shorter 5–6 s sprints (Metcalfe et al., 2022). Similar results were found when protocols with different sprint durations and repetitions matched for sprint volume were compared, suggesting that shorter sprints with more repetitions are perceived as more enjoyable and lead to greater intentions to engage in SIT (Townsend et al., 2017).

### 4.2 Number of bouts

To reduce the overall time commitment of interval-based exercise, several studies examined the effects of a reduced number of bouts performed (Metcalfe et al., 2016; Ramos et al., 2017; Nalçakan et al., 2018). In a recent acute study (McCarthy et al., 2024), scientists decreased the volume of SIT by reducing the number of 15-s bouts, leading to no difference in postexercise metabolism between protocols, suggesting that 1-min SIT (4 × 15 s) seems to be a viable option for elevated post-exercise V̇O_2_ and fat oxidation. However, from training studies, it appears that there is a threshold for the minimum number of sprint bouts per week that need to be performed to induce adaptations. In one study (Songsorn et al., 2016), inactive adults performed a single 20 s Wingate sprint 3 days per week for 4 weeks, and results showed this is an insufficient stimulus for improving VO_2_max. Similarly, performing a single 30-s all-out sprint 5 days per week for 6 weeks, failed to improve aerobic fitness and cardiometabolic factors in physically active individuals (Wong et al., 2024). The meta-regression conducted in a recent review of SIT studies demonstrated a significantly greater effect on performance-based outcomes with a higher number of sprints performed per session (Hall et al., 2023). On the contrary, another meta-analysis presented that increasing the number of sprint repetitions may actually decrease the improvement in VO_2_max, although the magnitude-based inference of this effect was ‘possibly small’ (Vollaard et al., 2017). The same authors further suggested that a reduced number of repetitions in SIT protocols may not compromise the increases in VO_2_max (Vollaard and Metcalfe, 2017). Furthermore, a recent meta-analysis demonstrated that during SIT, affect decreases progressively and proportionally with each additional sprint repetition (Metcalfe et al., 2022).

On the other hand, other studies using HIIT showed that as little as one bout of 4 min at 90% HRpeak, performed three times per week for 16 weeks, is sufficient to significantly ameliorate the severity of metabolic syndrome (Ramos et al., 2017), and to improve VO_2_max, and reduce blood pressure and fasting glucose in previously inactive adults after 10 weeks (Tjønna et al., 2013). These discrepancies may occur due to differences in participant fitness status, training duration, or interval volume used across studies.

### 4.3 Session and training duration

Regarding the effects of session duration on VO_2_max, previous meta-analyses stated that beneficial effects can be accrued even with a low dose of high-intensity exercise (≤5 min) (Wen et al., 2019; Yin et al., 2023), however, larger effects were demonstrated with an increased exercise time (≥15 min) (Xu et al., 2019) and total session duration (≥16 min) (Wang et al., 2021). A recent umbrella review with meta-analysis presented that HIIT protocols with low volume (i.e., <15 min of high-intensity exercise per session) yielded comparable effects for most body composition outcomes and possibly superior improvements in body fat reduction, compared with interventions with high-volume protocols. Furthermore, ≤5 min high-intensity exercise time was also found to improve cardiometabolic health (MetS z-score) and body composition (fat mass and waist circumference) (Yin et al., 2023).

The duration of the majority of HIIT/SIT studies typically ranges from 2 to 16 weeks (Batacan et al., 2017), even though a 5-year randomized controlled trial involving HIIT in older adults was recently published (Stensvold et al., 2020). In terms of the impact of training duration on cardiorespiratory adaptations, intense interval exercise training has been found to improve VO_2_max over short intervention periods (<4 weeks) (Wen et al., 2019), with other meta-analyses demonstrating that longer training durations (≥4–12 weeks) are associated with larger effects on VO_2_max compared to shorter interventions (Batacan et al., 2017; Wen et al., 2019; Wang et al., 2021). In contrast, the meta-analysis by Edwards et al. (2023) showed no effect of training duration on VO_2_max improvements. Nevertheless, the training duration will likely affect the kinetics at which central and peripheral adaptations occur to improve VO_2_max. For instance, central hemodynamic factors (i.e., blood volume) and peripheral processes involved in oxygen utilization, such as mitochondrial capacity may increase during the first weeks of training (Little et al., 2011; Mandić et al., 2022), while cardiac factors like peak cardiac output appear to manifest following longer training periods (Daussin et al., 2008; Raleigh et al., 2018; Bostad et al., 2021). On the other hand, some studies suggest that a rapid increase in VO_2_max occurs during the initial weeks of training, which subsequently reaches a plateau despite the continuation of the same training regimen (Yamagishi and Babraj, 2017; Lloria-Varella et al., 2024). Based on this conclusion, it could be argued that intense interval exercise training can be used at the beginning of a program as a “booster” before other forms of training are introduced.

Regarding metabolic adaptations, it appears that increased fat oxidation occurs rapidly, as studies reported this adaptation after as little as six sessions of SIT (Astorino et al., 2011) and HIIT (Talanian et al., 2010).

Similarly, insulin sensitivity has been demonstrated to improve following 2 weeks of SIT (Richards et al., 2010) and HIIT (Hood et al., 2011). Some potential skeletal muscle characteristics that have been previously observed following short-term HIIT/SIT could drive this response such as increased GLUT4, upregulated aerobic enzyme activity, and increased mitochondrial biogenesis (Talanian et al., 2010; Hood et al., 2011; Little et al., 2011). Notwithstanding, results from meta-analyses show that training durations that are moderate in length (≥8 weeks) are required for improving postprandial glucose (Khalafi et al., 2022) and are more beneficial for body fat%, fat, and fat-free mass than shorter-duration programs (Batacan et al., 2017; Khodadadi et al., 2023). Furthermore, the subgroup analysis of a recent umbrella review with meta-analysis revealed greater body fat reduction in longer-duration (≥12 weeks) interventions in apparently healthy adults, while this benefit appeared to be more prominent in individuals with overweight/obesity (Poon E. T.-C. et al., 2024).

### 4.4 Conclusion for manipulating time

Besides intensity, alterations in the duration of the interval and recovery periods, their time structure, the number of bouts, as well as the total training duration make it difficult to analyze responses and adaptations broadly. When comparing HIIT protocols with identical intensity and total work yet manipulated bout duration, there is a disparate cardiorespiratory and muscle metabolic response between the longer and shorter protocols that may elicit dissimilar central and peripheral adaptations to HIIT. Thus, the metabolic and cardiorespiratory strain can be adjusted according to each participant’s physical fitness and training goals. Furthermore, given that a reduction in bout duration during HIIT results in more positive affective responses, shorter bouts of HIIT are more appropriate when prescribing interval exercise to unfit, overweight adults or individuals with cardiorespiratory or metabolic diseases to promote adherence and future engagement to exercise.

## 5 Type

A variety of intense interval exercise training modalities have emerged throughout the years, with two general categories having arisen. One category is referred to as ‘‘unimodal training’’ which uses traditional aerobic exercise modalities such as running and cycling. Alternatively, there is the ‘‘multimodal training’’ which uses exercises with body weight, weighted objects, bars, or devices to perform circuit-type training. A relatively new multi-modal iteration is high-intensity functional training (HIFT), which combines both aerobic and muscle-strengthening exercises executed at high intensity with varying bout durations and recovery intervals. In the literature, investigators have used several terms to identify this type of training such as bodyweight high-intensity interval training, circuit training, resistance circuit training and Cross- Fit®. While HIFT and HIIT share some conceptual commonalities, such as the high-intensity nature of the bouts, there are distinct dissimilarities in their methodologies (i.e., the use of functional movement patterns and resistance-based exercises, or the exclusion of a defined rest interval in some cases), that result in important differences in physiological responses and adaptations (Feito et al., 2018a).

### 5.1 Unimodal-HIIT/SIT formats

The majority of data concerning responses to acute or chronic intense interval exercise was obtained from leg cycling or treadmill running as exercise modalities. Several other exercise types have been used in studies, although to a lesser extent, such as rowing, stair climbing, elliptical, arm cycling, jumping, or even sport-specific training (i.e., cross-country ski, etc.) (Fex et al., 2015; Allison et al., 2017; Karlsen et al., 2020; Astorino and Emma, 2021; Astorino et al., 2023; Venegas-Carro et al., 2023).

#### 5.1.1 Cardiorespiratory responses and adaptations

Several acute studies have compared the cardiorespiratory responses of different HIIT/SIT types. The study by Bogdanis et al. (2021) compared running vs. cycling high-intensity interval exercise protocols that consisted of 10 x 1-min efforts at 20% above the ventilatory threshold and showed that interval-based running imposes a greater cardiopulmonary stimulus compared with cycling as indicated by the higher HR and VO_2_ responses. Similarly, absolute mean VO_2_, HR as well as total time at > 90% VO_2_max were higher in running than cycling HIIT when male team sport players completed 3 × 6 min (with 5 min between sets) 1:1 efforts of 15 s bouts at 120% VO_2_max speed or power at VO_2_max on a treadmill or cycle ergometer, respectively (Twist et al., 2023). In another study comparing 10 × 1 min bouts of arm or leg cycling at 75 %PPO, it was demonstrated that leg cycling elicits higher relative mean HR than arm cycling with no difference in peak VO_2_ and HR (Astorino and Emma, 2021).

Various HIIT/SIT modalities have been found to be effective for improving cardiorespiratory fitness following training such as running (Venegas-Carro et al., 2023), cycling (Metcalfe et al., 2016; Stavrinou et al., 2018), stair climbing (Allison et al., 2017), elliptical (Fex et al., 2015), or combined aerobic modalities (Petersen et al., 2022). However, meta-analyses in the general population suggest that there is significantly greater improvement in VO_2max_ in response to walking/running HIIT protocols compared with cycling (Mattioni Maturana et al., 2021; Edwards et al., 2023). These differences can be explained by the greater capacity and requirement for oxygen consumption during running (Twist et al., 2023). Furthermore, compared to cycling, running has a faster oxygen uptake kinetic response (Hill et al., 2003). This larger and faster primary phase of the VO_2_ response may lead to larger time spent above 90% VO_2_max (Twist et al., 2023), which is considered a key parameter for eliciting maximal cardiorespiratory adaptations (Buchheit and Laursen, 2013).

#### 5.1.2 Changes in metabolic indices and body composition

Various studies have shown that dissimilar HIIT/SIT modalities can improve metabolic indices and body composition (Fex et al., 2015; Stavrinou et al., 2018; Petersen et al., 2022). An 8-week training study using a combination of two non-weight-bearing HIIT modalities, rowing and cycling, demonstrated significant increases in insulin sensitivity in obese men with and without type 2 diabetes (Petersen et al., 2022). These results suggest that the recruitment of several muscle groups may potentiate the insulin-sensitizing effect of exercise training. Furthermore, meta-analyses comparing the effectiveness of running vs. cycling on body composition showed that the reductions in body mass, body fat and visceral fat mass are greater following running HIIT compared with cycling HIIT (Wewege et al., 2017; Maillard et al., 2018) in overweight and obese participants. Interestingly, the mode of running can also impact the training response, since a subgroup analysis in a recent meta-analysis showed that overground running induced the highest reduction in %body fat and fat mass compared to treadmill running and cycling, probably due to activation of larger and more muscle groups (Khodadadi et al., 2023), and to the accelerations and decelerations inherent in shuttle running that would increase the metabolic cost of exercise (Stevens et al., 2015). In a recent study comparing cycling and running isoenergetic HIIT programs in men with overweight/obesity, the results showed that both programs improved body composition, while greater abdominal fat mass loss was found in the HIIT running group (Couvert et al., 2024). On the contrary, in a recent umbrella review, cycling appeared to be more efficacious than running/walking/jogging in reducing % body fat in apparently healthy adults (Poon E. T.-C. et al., 2024). One possible explanation is that cycling is a non-weight-bearing activity that is gentler on the joints, enabling thus individuals to sustain longer and more intense exercise sessions, leading to more efficient fat loss (Poon E. T.-C. et al., 2024). Furthermore, fat-free mass increased only in studies utilizing HIIT cycling, while treadmill running and overground running did not increase fat-free mass (Khodadadi et al., 2023). This discrepancy is mainly due to muscle hypertrophy of the lower extremities (Tsirigkakis et al., 2021) caused by the high forces exerted by the leg muscles during HIIT on a cycle ergometer.

Physiological and mechanical differences between the exercise modalities may account for the differences in metabolic responses and body composition. In acute HIIT studies, blood lactate concentration is higher in cycling than running (Bogdanis et al., 2021) and rowing (Clausen and Astorino, 2024), indicating that exercise involving a smaller muscle mass increases the metabolic strain and induces greater carbohydrate utilization. On the other hand, more muscle mass recruitment during running for any given sub-maximal workload relative to maximal capacity may presumably lead to greater energy expenditure (Millet et al., 2009). It has also been suggested that a greater fast twitch motor unit recruitment occurs during cycling compared to running due to the different muscle contraction regimens (i.e., concentric vs eccentric) (Capostagno and Bosch, 2010). Previous studies have shown that fat oxidation is significantly higher during running than during cycling at the same relative intensity (Capostagno and Bosch, 2010; Chenevière et al., 2010) and therefore, the relative contribution of carbohydrates to the total energy expenditure is higher in cycling; whereas, the relative contribution of fat is higher in running at high exercise intensities (Chenevière et al., 2010). However, the small number of training studies that compared the effects of running intense interval training with cycling on changes in fat oxidation does not permit further analysis (Atakan et al., 2022).

#### 5.1.3 Psychological responses

The increased blood lactate concentration induced by cycling would be expected to evoke higher RPE and more aversive affective responses given that blood lactate concentration has been associated with a negative change in affect during high-intensity interval exercise (Astorino and Vella, 2018). However, an acute study comparing running and cycling HIIT showed that post-exercise RPE, affective valence, and enjoyment were similar across modalities, despite the distinct physiological and metabolic responses (Bogdanis et al., 2021). Exercise modality alters localized perceived effort, with running eliciting higher central (breathlessness RPE) yet cycling higher peripheral sensations (leg-muscle exertion) (Twist et al., 2023).

### 5.2 High-intensity functional training

HIFT has been defined as a training style that incorporates functional, multimodal movements, performed at relatively high intensity, and designed to improve parameters of general physical fitness and performance (Feito et al., 2018a). The breadth of this definition may create various training protocols, making it difficult to generate results from studies and to interpret them in practice. Training characteristics such as the load, intensity, number of exercises, repetitions, sets and rounds, as well as recovery duration, allow a different dosage of training, and consequently, they may promote different endurance and strength-related adaptations (Ramos-Campo et al., 2021). The physiological, metabolic, and perceptual responses to HIFT are largely influenced by workout structure, primarily by exercise selection and the presence or absence of structured recovery intervals. This is particularly evident in CrossFit® workouts, where minimal recovery occurs between exercises. (McDougle et al., 2023).

#### 5.2.1 Cardiorespiratory responses and adaptations

Despite large variations in protocol characteristics, HR responses typically lie within the “vigorous-intensity” category (i.e., 77%–95% of HRmax) (McDougle et al., 2023). It was recently shown that a HIFT program including four rounds of a 9-exercise circuit with 30 s exercise and 15 s recovery can impose a near-maximal load on the cardiorespiratory system (95% of HRmax), while the average HR surpasses 90% HRmax for 50%–65% of the entire session (Posnakidis et al., 2022a). Thus, even though HIFT differs from unimodal training formats aimed to improve aerobic capacity such as running and cycling, aspects of its prescription provide adequate stimuli for aerobic adaptation leading to increased cardiorespiratory fitness (Ramos-Campo et al., 2021; Posnakidis et al., 2022a).

#### 5.2.2 Changes in metabolic indices and body composition

Given the current evidence, HIFT may elicit significant changes in body composition. Regarding fat mass, a meta-analysis showed that resistance circuit-based training performed at low or moderate intensities with a higher training volume (i.e., more repetitions and shorter rest time) may increase the magnitude of change in fat loss, with no change in body mass (Ramos-Campo et al., 2021). In accordance with these findings, in a recent study, body fat decreased equally in two groups in response to 6 weeks of HIFT with either low (30% 1-RM) or moderate load (70% 1-RM) of equal volume (Kapsis et al., 2022). However, after 12 weeks, body fat further decreased only in the low load group compared to moderate load, possibly due to the lower loads of resistance training coupled with a higher number of repetitions.

An increase in lean body mass was found in some (Murawska-Cialowicz et al., 2015; DeBlauw et al., 2021; Kapsis et al., 2022; Ameur et al., 2024) but not all studies (Fealy et al., 2018; Feito et al., 2018b; Posnakidis et al., 2022a). The discrepancies in the studies can be attributed to the differences in the intensity of the program and to the training status of the participants. Even though muscle hypertrophy can be equally achieved across a spectrum of loading ranges (Schoenfeld et al., 2017), it was suggested that the inclusion of some exercises with high load can stimulate muscle hypertrophy (Posnakidis et al., 2022b). The potential changes in lean body mass, in addition to possibly enhanced neural adaptations, may cause improvement in muscle strength, a result presented in many HIFT studies (Feito et al., 2018b; Ramos-Campo et al., 2021; Kapsis et al., 2022; Posnakidis et al., 2022b). From resistance training studies, it is known that maximal strength benefits are obtained from the use of heavy loads (Schoenfeld et al., 2017). In line with this, changes in upper body maximal strength were more substantial when high-load resistance exercises were added to a HIFT program (Posnakidis et al., 2022b). In contrast, in another study in young, physically active individuals, 12 weeks of HIFT using either low or medium load were found to be equally effective in improving maximal strength (Kapsis et al., 2022).

HIFT may also confer positive effects on metabolic dysfunction (Fealy et al., 2018; Smith et al., 2023; Ameur et al., 2024). Ameliorated insulin sensitivity, improved fat oxidation, and reduced cardiometabolic risk in individuals with type 2 diabetes were found after 6 weeks of a HIFT program including short 8–20 min high-intensity workouts at >85% HRmax (Fealy et al., 2018), with a concomitant β-cell function improvement (Nieuwoudt et al., 2017). Recently, improved lipid profile, glucose regulation, and body composition in inactive obese women occurred following 12 weeks of HIFT (Ameur et al., 2024). Interestingly, a recent study attempted to elucidate a minimal weekly HIFT frequency dose for cardiometabolic health in adults with metabolic syndrome (Smith et al., 2023). These preliminary results showed clinically meaningful improvements in the metabolic syndrome severity score with no effect of HIFT frequency after 12 weeks. Therefore HIFT, similar to HIIT (Stavrinou et al., 2018), represents a potential strategy to improve cardiometabolic health even at a low weekly dose.

#### 5.2.3 Psychological responses

HIFT has been shown to be an enjoyable exercise modality in physically inactive men and women (Smith et al., 2023), demonstrating significantly higher enjoyment compared with traditional moderate-intensity aerobic and resistance training (Heinrich et al., 2014), with the participants in both studies stating an intention to continue performing this modality. Moreover, in another study, HIFT resulted in equally positive affective responses (i.e., increased arousal and pleasure) compared with moderate continuous training and HIIT (Heinrich et al., 2020). Further, in contrast to HIIT, the slope of the affective response to HIFT continued to increase in pleasure and arousal until the end of the workouts, with the authors suggesting that these affective responses may be due to the ability of participants to self-regulate their level of effort within HIFT. The variations in training design and characteristics of HIFT may lessen the potential monotonous nature of the repetitive aerobic formats of HIIT. During high-intensity interval resistance training, overall enjoyment was favored in self-selected compared to fixed recovery intervals, even though affective perception was not influenced by the strategy of recovery intervals (Fidalgo et al., 2023). Finally, it has been suggested that because HIFT classes are usually held in a group setting, they may develop, among others, a sense of community and stimulate competitiveness which are related to increased motivation and adherence (Dominski et al., 2021). However, more studies are needed to evaluate these perceptual responses throughout a long-term HIFT program.

### 5.3 Conclusion for manipulating type

The choice of intense interval exercise training type can profoundly affect the training adaptations. Unimodal modalities recruiting a large muscle mass including running or rowing could be a more efficient strategy to elicit cardiorespiratory improvement and possibly decrease fat-mass deposits, without compromising the positive perceptual responses, compared to cycling-based HIIT. Regarding the occurrence of adverse events or rates of adherence, no difference between cycle and treadmill-based HIIT regimens was found in a meta-review (Martland et al., 2020). However, the overall safety and sustainability of implementing running for overweight/obese populations or individuals with joint pain due to increased impact load is limited (Wewege et al., 2017). Taking into consideration that dropout rates were found to be significantly lower in cycling-based interventions compared with studies using running/walking as an exercise modality and that most injuries occurred in studies using running/walking (Reljic et al., 2019), non-weight-bearing unimodal HIIT/SIT protocols could be a safe and effective alternative to maximize tolerability and reduce the risk of injuries (Petersen et al., 2022). Furthermore, given the current evidence, it appears that HIFT may also be a viable and effective approach to obtain cardiorespiratory and metabolic benefits, favorable body composition changes, and induce positive perceptual responses. The manipulation of the workout characteristics creates large variations in the design of a HIFT program, which can be tailored according to the needs and abilities of each individual.

## 6 Periodization

The principles of exercise training state that for optimal results and continuous improvements in physical fitness through an individual’s participation in an exercise program, the program must change at regular intervals, the intensity must progressively increase, and there should possibly be a variation in the exercises. However, many studies investigating intense interval exercise training offer no variation or progression in the training stimulus. When interval training is performed intensely without adequate recovery, it may induce adverse effects that can impact various metabolic (Tsirigkakis et al., 2022), endocrine, and immune (Athanasiou et al., 2023) responses, as well as perceptual and affective responses (Tsirigkakis et al., 2022). For instance, one study in obese individuals comparing two work-matched HIIT programs with repeated short (10s) or long (60s) bouts revealed no difference in blood lactate concentration response and RPE, and more aversive responses in response to longer bouts following 8 weeks of training (Tsirigkakis et al., 2022). This lack of metabolic adaptation together with the reduced affective responses during training, suggests the possibility of an overreaching response and a reduced adherence with longer bouts. An overreaching effect was also assumed in another HIIT study using four to six high-intensity 30-s bouts in young adults (Bogdanis et al., 2022b). Results showed that cortisol concentration was significantly decreased for up to 48 h after the final HIIT session following 3 weeks of training compared to baseline (Bogdanis et al., 2022b), even though there are conflicting results regarding the relationship between cortisol responses and overreaching (Cadegiani and Kater, 2017). The same study also showed a significant decrease in lymphocyte responses after training which may indicate lymphocyte apoptosis leading to immunosuppression, a potential index of overreaching and overtraining syndrome (Meeusen et al., 2013). Another study in healthy young adults showed that performing intermittent running to exhaustion for three consecutive days may impair immune restoration and exacerbate lymphocyte migration and apoptosis, leading to reduced immunity (Navalta et al., 2014). The study by Flockhart et al. (2021) investigated the dose-response relationship of exercise training and demonstrated that excessive HIIT caused mitochondrial impairment and reduced glucose tolerance in recreationally active volunteers. The authors suggest that there is an upper limit to the amount of intensive exercise that can be performed without disrupting metabolic homeostasis, while beyond this limit, negative effects on metabolic health and adaptation of physical performance start to manifest.

Considering thus the high physiological demand of intense interval exercise training, training periodization, i.e., the planned intense interval exercise training manipulation of the four components of F.I.T.T., is essential for optimizing physiological adaptations and performance and concomitantly minimizing the risk of excessive training and maladaptations. Block periodization of HIIT (including defined blocks of increased focus on specific intensities), has been used in endurance athletes (Karlsen et al., 2020) providing superior adaptations than traditional organization (Rønnestad et al., 2014). Furthermore, the HIIT shock microcycle appears as a viable strategy to quickly improve endurance parameters and performance in athletes. In these blocks, a congested distribution of HIIT sessions is implemented (at least two HIIT sessions every 3 days for periods ranging from 7 to 21 days to provide high stimuli for endurance adaptation) followed by 5–7 days of recovery to dissipate the fatigue induced by the density of HIIT sessions within a short timeframe (Dolci et al., 2020). Nevertheless, because the design of intense interval exercise training protocols in athletes is beyond the scope of this review, interested readers may have a deeper insight from exploring previously published reviews (Buchheit and Laursen, 2013; Dolci et al., 2020).

Less studies have been published involving intense interval exercise training periodization in non-athletic populations. Active adults performed 6 weeks of three different HIIT regimes characterized by a modification of intensity duration, and overall structure (Astorino et al., 2017). The results showed that the magnitude of increase in VO_2_max was similar across regimes and was mediated by significant increases in maximal cardiac output, however, the time course of change differed across groups. In another study, multiple sclerosis patients were randomized into a 12-week periodized HIIT (including 3 × 20 s all-out sprints and classic endurance training) or a classic endurance training program (Keytsman et al., 2021). The study found that periodized HIIT training is well-tolerated in this population and that despite a substantially reduced training volume (−57%), induced larger improvements in exercise capacity compared to classic endurance training. Therefore, in a real-world environment, to obtain greater long-term improvements, intense interval exercise training should be periodized and combined with moderate-intensity continuous exercise around or below the lactate threshold, as well as with resistance exercise. Furthermore, by modifying bout duration, the acute metabolic and cardiorespiratory strain can be adjusted according to one’s physical fitness and training goals, fine-tuning training load progression and enabling periodization of HIIT. Nevertheless, the optimal training intensity distribution in non-athletic populations warrants further investigation.

## 7 Conclusion

Intense interval exercise training is a highly effective approach for promoting various health and performance benefits. The creation of an effective training program is determined by complex interactions between training frequency, intensity, time and type, producing multiple ways to achieve optimal responses and adaptations. Given the complexity and interrelated nature of the training variables, progression could be achieved through many different approaches. The duration of exercise bout and recovery interval are critical for the manipulation of almost all acute responses to HIIT, enabling periodization of HIIT, and promoting optimal adaptations and exercise adherence. In addition, a considerable level of adaptations may be achieved with training frequencies as low as once or twice per week, adding to the feasibility of this exercise mode.

It is important to note that physiological, metabolic and perceptual responses to different intense interval exercise protocols can vary depending on the population being studied. For example, sedentary individuals are likely to experience more negative affect compared to active individuals when performing the same protocols. Therefore, to enhance exercise effectiveness and adherence, it may be more beneficial for sedentary individuals to engage in protocols that elicit more positive perceptual responses (i.e., shorter bouts). In general, as individuals may have different exercise-related goals, abilities, and preferences, a customized intense interval exercise training program can be designed based on the proper manipulation of the F.I.T.T. components. This may turn intense interval exercise training into an attainable, pleasant, safe and effective mode of exercise which can be performed without the need of specialized equipment. However, further research is needed to improve our understanding of how to manipulate these components optimally.
